# Metabolic Syndrome and Hemorrhagic Stroke in Hispanic Elderly Patients with Cerebral Cavernous Malformations

**DOI:** 10.3390/diagnostics15091144

**Published:** 2025-04-30

**Authors:** Alok K. Dwivedi, David Jang, Ofek Belkin, Justin Aickareth, Mellisa Renteria, Majd Hawwar, Jacob Croft, M Ammar Kalas, Marc Zuckerman, Jun Zhang

**Affiliations:** 1 Departments of Molecular and Translational Medicine, Texas Tech University Health Sciences Center, El Paso, TX 79905, USA; alok.dwivedi@ttuhsc.edu (A.K.D.);; 2 Departments of Internal Medicine, Texas Tech University Health Sciences Center, El Paso, TX 79905, USA

**Keywords:** cerebral cavernous malformations (CCMs), metabolic syndrome, risk factors, hemorrhagic stroke, Mexican Hispanics

## Abstract

**Background/Objectives:** Cerebral cavernous malformations (CCMs) are neurological disorders that increase the risk of hemorrhagic stroke. The Mexican Hispanic population has a higher prevalence of both CCMs and metabolic syndrome (MetS), defined by the presence of three or more of the following: central obesity, elevated triglycerides, low HDL, dyslipidemia, hypertension, or elevated fasting glucose. MetS is also associated with an increased risk of hemorrhagic stroke. However, the connection between MetS and hemorrhagic stroke in Hispanic CCM patients remains uncertain. Additionally, it is unclear if Hispanic CCM patients have different cardiometabolic profiles compared to controls. **Methods:** We analyzed a retrospective cohort of Mexican Hispanic adult CCM patients, including age- and gender-matched controls from the NHANES database. Fisher’s exact test or an unpaired Student’s *t*-test was used to compare risk factors between the CCM cohort and controls. Additionally, we conducted relative risk regression analysis to assess the adjusted association of MetS with hemorrhagic stroke. **Results:** The CCM cohort showed higher rates of epilepsy (24.6% vs. 1.6%, *p* < 0.001) and hemorrhagic stroke (36.6% vs. 3.6%, *p* < 0.001), but a lower prevalence of MetS (14% vs. 54.8%, *p* < 0.001) compared to age- and gender-matched Mexican Hispanic controls. The adjusted analysis revealed that among CCM patients in the older age group (age ≥ 50 years), MetS was associated with hemorrhagic stroke (RR = 2.38, 95%CI: 1.40–4.02, *p* = 0.001). **Conclusions:** This study reveals distinct features of CCMs in the Mexican Hispanic population, indicating a higher risk of hemorrhagic stroke and epilepsy compared to other ethnic groups. To mitigate the risk of hemorrhagic stroke, controlling blood pressure and managing comorbidities like metabolic syndrome (MetS) and epilepsy are essential, particularly in CCM patients aged 50 years and above.

## 1. Introduction

Cerebral cavernous malformations (CCMs) are neurological disorders characterized by the abnormal dilation of intracranial capillaries in the brain, which increases susceptibility to hemorrhagic stroke [1]. As an inherited condition, familial cases of CCMs (fCCMs) have been linked to three specific genes, CCM1, CCM2, and CCM3 [2,3], while the causation of sporadic cases of CCMs (sCCMs) is still under active investigation [4,5]. Hemorrhagic CCMs are often caused by defects in the blood–brain barrier (BBB), which can result in microvessel rupture [6,7]. Accumulated genetic data have shown similar mutation rates across all ethnic groups, with variable penetrance, leading to many carriers of the CCM gene mutation who do not exhibit clinical consequences. However, for reasons that remain unclear, the Hispanic population experiences a higher prevalence of symptomatic CCMs with more severe health outcomes, potentially due to unidentified genetic or environmental triggers. This study is significant not only because of the increased burden of mortality, disability, and costs associated with hemorrhagic stroke in this population, but also for its potential to uncover unknown risk factors that contribute to the pathogenesis of CCMs [8]. Moreover, the Hispanic population is a heterogeneous group and represents the different prevalence of risk factors according to Hispanic origin. Mexican Hispanics, particularly those living on the US–Mexico border, have a higher prevalence of metabolic abnormalities and diabetes, predisposing them to a greater risk for cardiovascular diseases, including stroke, compared to other Hispanic and non-Hispanic white populations. Therefore, it is critical to estimate the prevalence of hemorrhagic stroke and other cardiometabolic factors in the Mexican Hispanic CCM cohort compared to Hispanic and non-Hispanic white controls.

Further research is needed to fully understand the primary risk factors for hemorrhagic stroke in Hispanics. However, based on current investigations, metabolic syndrome (MetS), defined by the presence of three or more of the following, central obesity, elevated triglycerides, low HDL, dyslipidemia, hypertension, or elevated fasting glucose, could be a potential contributing factor [9]. Evidence indicated that fCCM patients with deficient CCMs proteins, specifically those with CCM1 gene deficiency, have exhibited disrupted metabolic function, which usually leads to oxidative stress and inflammatory response [10,11,12,13]. Developmental venous anomalies (DVAs), which are highly associated with sCCMs [14,15,16,17], have also been linked to MetS [18,19,20]. However, only a few, inconsistent results have been published regarding the association between MetS and hemorrhagic CCMs [21,22]. Obesity was found to be a significant risk factor for sCCM hemorrhagic events in a large European sCCM cohort [21], while fCCM hemorrhage was only borderline associated with obesity and systolic blood pressure (SBP) in a large Mexican Hispanic population [22]. However, it is unclear whether MetS or SBP is associated with hemorrhagic stroke among CCM patients or not after adjusting for obesity. This study aimed to assess the prevalence of hemorrhagic stroke and its primary risk factors in Mexican Hispanic CCM patients compared to Hispanic and non-Hispanic white individuals without CCM.

## 2. Materials and Methods

*Study population.* This retrospective study was conducted using the El Paso CCM Retrospective Study Part 1 database, which includes 184 symptomatic CCM subjects identified through a retrospective chart review. The review included patients with ICD-9/10 diagnosis codes covering the CCM clinical spectrum (Supplementary Table S1), familial history, and CT and MRI diagnostics from 2010 to 2023. CCM diagnoses made between 2010 and 2023 were included, with data collected from the Texas Tech University Health Sciences Center El Paso clinics and the University Medical Center, El Paso (UMC). This study was conducted in accordance with an approved protocol (E22031) from the institutional review board (IRB) at Texas Tech University Health Sciences Center El Paso (TTUHSCEP). We only included adult CCM patients with self-reported ethnicity of Mexican Hispanic. We also retrospectively obtained variables of interest from patient charts for both familial (fCCMs) and sporadic cases (sCCMs) of CCMs registered at TTUHSCEP/UMC. The physicians’ diagnoses and research staff followed the inclusion criteria for symptomatic CCMs.

*Data acquisition.* Data were collected by six trained researchers, mostly medical students supervised by medical residents or postdoctoral fellows. An independent researcher entered the data into the database for standardization and quality control. The selected CCM subjects in the database were all symptomatic CCM subjects from 2010 to 2023 from the El Paso border region, with a complete medical history and of non-Hispanic White or Mexican Hispanic ethnicity.

Inclusion and exclusion of subjects: the database was thoroughly examined for inconsistencies and missing data. Any discrepancies or missing data points were cross-checked with the original data collection sheet or the medical record. Subjects with inconsistent records were excluded (Supplementary Table S1). Data that were unavailable were considered as missing, and subjects were presumed not to have the condition unless it was explicitly noted in their medical chart. After completing this process, the database was reviewed again, and subjects were excluded based on specific criteria, including incomplete medical records, traumatic or infectious stroke etiology, post-operative stroke etiology, presence of brain tumors, lack of radiographic evidence of infarction, Hispanic birthplace outside the border region (not of Mexican descent), or origins other than non-Hispanic White or Mexican Hispanic ethnicity (such as Asian, Black, Native American, or White). The final dataset included only symptomatic CCM subjects with complete medical histories and of non-Hispanic White or Mexican Hispanic descent.

Outcome: the primary outcome of this study was a hemorrhagic stroke, confirmed based on the ICD-9 or 10 codes included in Supplementary Table S1. We included any hemorrhagic stroke diagnosis among individuals with CCM mutations throughout the study duration. Exposure and risk factors: the primary exposure of interest was MetS, which was defined as the presence of three or more of the following: central obesity, elevated triglycerides, reduced high-density lipoprotein (HDL), dyslipidemia, systemic hypertension, or elevated fasting glucose [23]. With this classification and well established MetS clinical diagnosis guidelines [24], subjects with MetS were identified in the El Paso CCM Retrospective Study Part 1 database. Some major parameters of MetS, such as SBP, diastolic blood pressure (DBP), blood glucose levels, and body mass index (BMI) were also included in this study. In addition to MetS, another critical risk factor included was epilepsy. Other covariates included age and sex. BMI was calculated using the standard formula in kg/m^2^.

Non-CCM controls: for the selection of non-CCM controls, we utilized the well-established, nationally representative National Health and Nutrition Examination Survey (NHANES) database. Specifically, we selected controls from the most recent publicly available pre-pandemic cycle (2017–2018) of NHANES. Initially, all Mexican Hispanics (*N* = 1367) and non-Hispanic whites (*N* = 3150) from this NHANES cycle were included. After excluding non-adults, 792 Mexican Hispanics and 2032 non-Hispanic whites remained eligible for control selection. A propensity score model was then developed to match each CCM subject with two age- and sex-matched controls (1:2 case-to-control selection). Therefore, we selected a total of 368 Mexican Hispanics and 368 non-Hispanic whites. We followed the exact definition of MetS as specified in the above section, and defined MetS using the NHANES-based calculations [25]. The NHANES collects data from participants on prescribed medications in the past 30 days. We defined epilepsy by whether participants had taken medications for “epilepsy and recurrent seizures”.

*Data analysis.* Baseline characteristics were summarized using appropriate summary measures, including mean and standard deviation (SD) for continuous variables and frequency and percentage for categorical variables. All the characteristics were compared according to the presence and absence of MetS using either Fisher’s exact test or unpaired Student’s *t*-test. The unadjusted and adjusted association of each factor with the presence of hemorrhagic stroke among the CCM cohort was primarily determined using relative risk regression analysis [26]. Multiple multivariable relative risk regression models were developed, including a model with MetS and a model with individual components of MetS. In the multivariable model, effect modifiers for the association between MetS and hemorrhagic stroke were explored. In the presence of a strong modifying effect of age, the final relative risk regression model was developed for patients with age ≥ 50 years.

Furthermore, the distribution of hemorrhagic stroke and risk factors between the Hispanic CCM cohort and the age- and sex-matched healthy controls were compared using appropriate statistical tests, such as Fisher’s exact test or unpaired Student’s *t*-test. The age- and sex-matched controls were observed using propensity scores logistic regression models, and multivariable logistic regression models were developed to identify differences in risk factors between the CCM cohort and controls. The results of relative risk regression analysis were presented with risk ratio (RR), 95% confidence interval (CI), and *p*-value, while the results of logistic regression models were summarized with the odds ratio (OR), 95% CI, and *p*-value. *p*-values less than 5% were considered statistically significant results. Z-standardized form of continuous variables were used in the regression analysis. The continuous variables in the regression analysis were z-standardized. Statistical analyses were carried out using STATA 17 (https://filecr.com/windows/stata-mp/ (accessed on 27 April 2025)). We followed the conduct and reporting of statistical analysis guidelines in medical research [27,28].

## 3. Results

**Figure 1:**
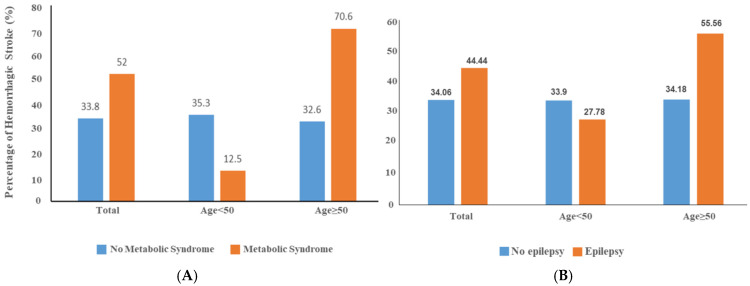
The prevalence of hemorrhagic stroke and epilepsy varies with metabolic syndrome (MetS) and age in the Hispanic CCM cohort. (**A**). Comparison of hemorrhagic stroke according to metabolic syndrome status by two age groups in Mexican Hispanic cerebral cavernous malformations (CCM) cohort. (**B**). Comparison of epilepsy according to metabolic syndrome status by two age groups in Mexican Hispanic cerebral cavernous malformations (CCM) cohort.

*Characteristics of the CCM cohort.* This study involved a Mexican Hispanic CCM cohort consisting of 184 participants. The average age of the cohort was 52.8 years (SD: 17.2), with 53% of the participants being female. Among the cohort, 36.6% (*n* = 67) had a hemorrhagic stroke, approximately 25% (*n* = 45) were diagnosed with epilepsy, and 14% (*n* = 25) had metabolic syndrome (MetS). In the CCM cohort, there were no significant differences in age, sex, or blood pressure between patients with and without MetS. As expected, patients with MetS had higher blood glucose levels and increased BMI (Table 1). The prevalence of hemorrhagic stroke was significantly higher in MetS patients than in those without MetS (70.6% vs. 32.6%), especially among older individuals. In contrast, among patients under 50 years of age, the trend was reversed (35.3% vs. 12.5%) (Figure 1A). A similar age-dependent pattern was seen in the epilepsy group among CCM individuals (Figure 1B), suggesting opposing genetic effects of CCM mutations in younger versus older carriers.

Unadjusted association of MetS and risk factors with hemorrhagic stroke in the cohort. In the unadjusted analysis, the presence of MetS (OR = 1.54, *p* = 0.053) and elevated SBP (OR = 1.31, *p* < 0.001) were associated with a higher risk of hemorrhagic stroke. Although epilepsy was not statistically significant (OR = 1.30, *p* = 0.19), it was also linked to an increased risk of hemorrhagic stroke (Table 2). We observed a strong interaction effect of age on the association between MetS (OR = 1.04, *p* = 0.007) and hemorrhagic stroke. MetS was found to increase the risk of hemorrhagic stroke in individuals aged ≥50 years (RR = 2.17, *p* < 0.001, Supplementary Table S2). However, there was no association between MetS or epilepsy and the risk of hemorrhagic stroke in younger CCM individuals (aged <35 years or 35–50 years) (Supplementary Table S3).

**Figure 2:**
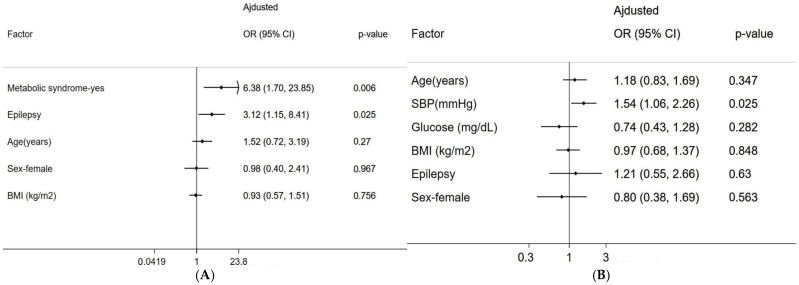
The association of metabolic syndrome with hemorrhagic stroke in Mexican Hispanic CCM patients by age groups. The significant association between the occurrence of hemorrhagic stroke and MetS and epilepsy in Mexican Hispanic CCM patients was only found in the older age group (≥50 years) (**A**), but not in younger age groups (**B**) after adjusting for other factors. Z-standardized form of continuous variables included in the regression analysis.

**Figure 3:**
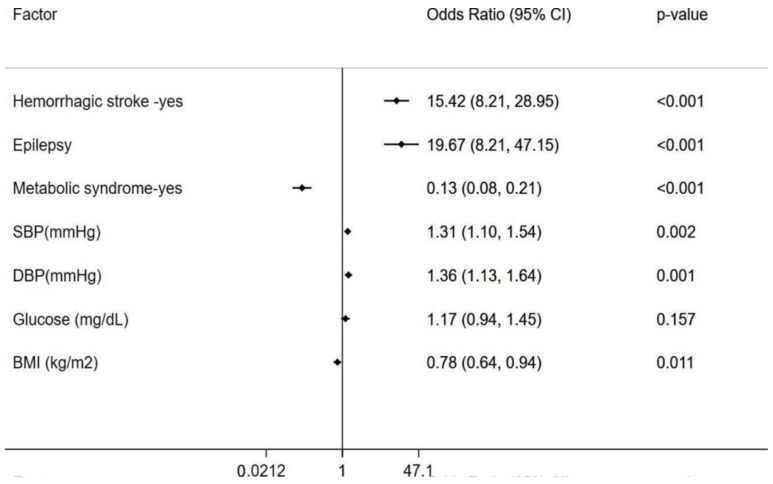
The association of individual components of metabolic syndrome with hemorrhagic stroke in Mexican Hispanic CCM patients. A significant association between the occurrence of hemorrhagic stroke and systolic (SBP) and diastolic (DBP) blood pressure in Mexican Hispanic CCM patients were found, after adjusting for other factors. Z-standardized form of continuous variables included in the regression analysis.

Unadjusted and adjusted associations of MetS and risk factors with hemorrhagic stroke in CCM Patients. In the unadjusted analysis of CCM patients, MetS (RR = 2.17, *p* < 0.001) and epilepsy (RR = 1.63, *p* = 0.037) were significantly associated with hemorrhagic stroke only in those aged ≥50 years, while blood pressure was linked to hemorrhagic stroke in individuals aged <50 years (Table 3). After adjusting for age, sex, and BMI, both epilepsy (RR = 1.77, 95% CI: 1.10–2.86, *p* = 0.019) and MetS (RR = 2.38, 95% CI: 1.40–4.02, *p* = 0.001) remained significant risk factors for hemorrhagic stroke among older CCM patients, but not in younger CCM patients (Figure 2). In the analysis of the individual components of MetS, both higher SBP and DBP were significantly associated with hemorrhagic stroke after adjusting for other cofactors (Figure 3).

Comparison of risk factors between the CCMs cohort and Mexican Hispanic Controls. When compared to age- and gender-matched Mexican Hispanic controls, the Mexican Hispanic CCM cases exhibited a significantly higher prevalence of epilepsy (1.6% vs. 24.6%, *p* < 0.001) and hemorrhagic stroke (3.6% vs. 36.6%, *p* < 0.001), but a lower prevalence of MetS (54.8% vs. 14%, *p* < 0.001). Among the individual components of MetS, CCM patients had elevated blood pressure levels (both SBP and DBP) but lower BMI compared to age- and gender-matched Hispanic healthy controls (Table 3). Similarly, when compared to non-Hispanic white controls, the Mexican Hispanic CCM cases had a significantly higher proportion of epilepsy (0.8% vs. 24.6%, *p* < 0.001), hemorrhagic stroke (4.8% vs. 36.6%, *p* < 0.001), and a lower prevalence of MetS (44.1% vs. 14%, *p* < 0.001). In terms of individual MetS components, Hispanic CCM patients had higher blood pressure and fasting glucose levels than non-Hispanic white controls (Figure 3).

## 4. Discussion

In this study, we sought to estimate the prevalence of hemorrhagic stroke and associated risk factors in the Mexican Hispanic CCM compared to Hispanic and non-Hispanic white non-CCM controls. We also investigated whether MetS or SBP is independently associated with hemorrhagic stroke in a cohort of Mexican Hispanic subjects with an admixture of sCCM and fCCM cases. By performing a comparative analysis of key components associated with MetS in this population compared to age- and gender-matched healthy individuals, we hoped to gain a better understanding of the association between the risk factors and hemorrhagic stroke.

CCMs are neurological conditions marked by the abnormal dilation of brain capillaries, which elevates the risk of hemorrhagic stroke [1]. Among ethnic groups, Mexican Hispanics demonstrate an earlier onset, higher incidence, and increased recurrence rates of hemorrhagic stroke, leading to more severe clinical manifestations of CCMs [8]. Understanding the risk factors for hemorrhagic stroke in this population is, therefore, essential. Our study found a significant association between epilepsy and hemorrhagic stroke in older CCM patients, with a higher prevalence of epilepsy compared to controls, highlighting their increased risk for related conditions. Furthermore, elevated blood pressure and glucose levels, rather than BMI, were identified as the primary risk factors in the CCM cohort when compared to non-CCM controls. The study firstly revealed that MetS and epilepsy were significantly associated with hemorrhagic stroke in CCM patients aged 50 years and older.

Metabolic syndrome (MetS) has long been associated with cardiovascular diseases [23,29,30,31]. Its association with ischemic stroke has been extensively studied across various ethnic groups [30,31], including the Mexican Hispanic population [32]. However, the relationship between MetS and hemorrhagic stroke remains less clear [33]. Evidence suggests that metabolic dysfunction and its impacts are linked to conditions associated with CCM deficiencies [10,11,12,13], particularly in cases involving CCM1 gene deficiency [10,13,34]. This points to a potential link between metabolic dysfunction and CCMs, though further research is necessary to fully elucidate this relationship. Our study suggests an association between MetS and hemorrhagic stroke in CCM patients, particularly among older age groups. Additionally, developmental venous anomalies (DVAs), which are often associated with sporadic CCMs, have also been connected to MetS [14,15,16,17,18,19,20]. A prospective study identified DVAs as a significant risk factor for hemorrhagic events in brainstem CCMs, indirectly supporting our findings [35]. However, a retrospective study involving a large cohort of sporadic CCM patients found obesity to be the only significant risk factor for hemorrhagic events, with no significant associations observed between other MetS components and CCM hemorrhage [21]. Unexamined interactions between age and MetS in previous CCM cohort analyses may explain the observed discrepancies. A separate study on familial CCM patients reported associations between hemorrhage and both obesity and systolic blood pressure (SBP), but not with diabetes or hyperlipidemia. Similarly, our study identified a significant independent association between SBP and hemorrhagic stroke in the CCM cohort, regardless of obesity or fasting glucose levels [22].

Our study identified a significant association between epilepsy and hemorrhagic stroke, particularly in older CCM patients, which is consistent with prior research highlighting this link in older adults [36]. Additionally, our CCM cohort showed a higher prevalence of epilepsy compared to controls, emphasizing their increased risk for epilepsy and related conditions. Post-stroke seizures and epilepsy are common in older adults, contributing to heightened metabolic stress and mortality [37]. In terms of metabolic syndrome (MetS), despite Hispanics generally having the highest MetS prevalence among ethnic groups, the CCM cohort displayed a lower prevalence of MetS compared to both Hispanic and non-Hispanic controls. This suggests that CCMs increase stroke risk by elevating hypertension and glucose levels rather than obesity. Elevated glucose and hypertension exacerbate oxidative stress and uric acid levels, impairing vascular relaxation by reducing nitric oxide and raising the risk of hemorrhagic stroke [38].

Like many other CCM studies, our study has certain limitations that should be taken into account. First, its cross-sectional design limits our ability to establish a definitive causal relationship between hemorrhagic strokes and MetS. Second, the retrospective nature of the study limited our ability to collect comprehensive data on various risk factors related to metabolic health and stroke. Thirdly, the study lacks information on the specific type of CCM or associated genetic mutations (familial or sporadic) which could offer additional scientific insights. Fourthly, although the overall cohort size is substantial, some subgroups, such as patients with MetS, had relatively smaller sample sizes after stratification. This limitation affects the statistical power and reliability of the conclusions drawn for these specific subgroups. Lastly, as a retrospective study, it inherently lacks the ability to definitively establish causation between risk factors and health conditions. While we utilized robust analytical tools to mitigate these limitations, the conclusions drawn may still be viewed as potentially overextended. Despite these limitations, our study represents one of the first efforts to characterize the relationship between hemorrhagic stroke, MetS, and epilepsy within a large, single-center cohort of Hispanic CCM subjects. Additionally, our group has joined an international consortium to conduct a multi-center study, which we believe will help address the aforementioned limitations of this study. Finally, it is worth highlighting that a key strength of this study lies in the inclusion of two age- and sex-matched ethnic control groups. This design enabled us to identify differences in CCM characteristics compared to non-CCM subjects, utilizing the largest single-center CCM cohort reported to date.

Our study on CCMs in the Mexican Hispanic population reveals several important findings. More than one-third of Mexican Hispanic CCM patients experienced hemorrhagic stroke, and one-quarter had epilepsy, indicating a higher risk of these conditions compared to other ethnic groups. Elevated systolic blood pressure was independently associated with hemorrhagic stroke in the CCM cohort. Among patients aged 50 and older, both metabolic syndrome and epilepsy were identified as significant risk factors for hemorrhagic stroke. Overall, the study highlights the need for managing blood pressure and addressing comorbidities such as MetS and epilepsy in CCM patients, particularly those over 50, to reduce hemorrhagic stroke risk. These results provide a foundation for future multicenter studies examining the potential benefits of monitoring blood pressure and managing MetS to reduce hemorrhagic stroke risk in CCM patients, especially older adults. As a genetically admixed population, findings on CCMs from Hispanic cohorts have been largely validated across other ethnic groups. Therefore, our study not only provides valuable insights into health disparities, but also contributes to understanding the potential pathophysiological mechanisms of CCM, as evidenced by early advancements in the field. These findings warrant further investigation in future research.

## Figures and Tables

**Table 1:** Distribution of characteristics of Hispanic CCM patients in the entire cohort and by metabolic syndrome (MetS). BMI: body mass index; SBP: systolic blood pressure; DBP: diastolic blood pressure; SD: standard deviation.

Parameter	Total Cohort	MetS-0 (No)	MetS-1 (Yes)	*p*-Value
Sample size (*N*)	184	154	25	
Age (years), mean (SD)	52.8 (17.2) (*n* = 184)	51.9 (17.4)	56.2 (14.9)	0.24
Sex				0.67
Male	86 (46.7%)	71 (46.1%)	13 (52.0%)	
Female	98 (53.3%)	83 (53.9%)	12 (48.0%)	
Systolic blood pressure (SBP), mean (SD)	134.3 (23.0) (*n* = 175)	132.4 (21.6)	139.6 (26.9)	0.14
Diastolic blood pressure (DBP), mean (SD)	76.0 (13.2) (*n* = 175)	75.7 (13.7)	76.2 (10.0)	0.85
Blood glucose levels, mean (SD)	135.3 (85.4) (*n* = 145)	126.2 (55.1)	178.7 (170.3)	0.007
BMI, mean (SD)	29.1 (6.2) (*n* = 169)	28.1 (5.2)	35.0 (8.2)	<0.001
Metabolic syndrome				
0 (No)	154 (83.7%)			
1 (Yes)	25 (13.6%)			
Missing	5 (2.7%)			
Epilepsy				0.63
0 (No)	138 (75.0%)	114 (74.0%)	20 (80.0%)	
1 (Yes)	45 (24.5%)	40 (26.0%)	5 (20.0%)	
Missing	1 (0.5%)			
Stroke				0.11
0 (No)	116 (63.0%)	102 (66.2%)	12 (48.0%)	
1 (Yes)	67 (36.4%)	52 (33.8%)	13 (52.0%)	
	1 (0.5%)			

**Table 2:** Association of covariates with hemorrhagic stroke status among Hispanic CCM patients. (A). Unadjusted association of covariates with hemorrhagic stroke status among Hispanic CCM patients. (B). Adjusted association of MetS with hemorrhagic stroke status among Hispanic CCM patients in older age group (≥50 years). BMI: body mass index; SBP: systolic blood pressure; DBP: diastolic blood pressure; MetS: Metabolic syndrome; SD: standard deviation; OR: odds ratio; CI: confidence interval.

Factor	No Stroke	Stroke	OR	95%CI		*p*-Value
Sample size (*N*)	116	67				
Age (years), mean (SD)	51.6 (16.3)	54.8 (18.8)	1.01	0.99	1.03	0.226
Gender						
Male	53 (45.7%)	33 (49.3%)	0.87	0.47	1.58	0.642
Female	63 (54.3%)	34 (50.7%)				
SBP, mean (SD)	129.9 (20.2)	140.8 (25.4)	1.02	1.01	1.04	0.003
DBP, mean (SD)	74.7 (11.3)	78.0 (15.7)	1.02	1.00	1.04	0.117
Blood glucose levels, mean (SD)	139.7 (106.1)	128.8 (46.5)	1.00	0.99	1.00	0.463
BMI, mean (SD)	29.1 (6.4)	29.1 (6.1)	1.00	0.95	1.05	0.989
Metabolic syndrome						
0	102 (89.5%)	52 (80.0%)				
1	12 (10.5%)	13 (20.0%)	2.13	0.91	4.99	0.083
Epilepsy						
0	91 (78.4%)	47 (70.1%)				
1	25 (21.6%)	20 (29.9%)	1.55	0.78	3.07	0.211

**Table 3:** Comparison of Hispanic CCM cohort with Hispanic controls from NHANES (2017–2018). BMI: body mass index; SBP: systolic blood pressure; DBP: diastolic blood pressure; SD: standard deviation; OR: odds ratio; CI: confidence interval; NHANES: National Health and Nutrition Examination Survey. * *p*-values < 0.05 for comparison between Mexican Hispanic CCM with Mexican Hispanic controls; ** *p*-values < 0.01 for comparison between Mexican Hispanic CCM with non-Hispanic white controls.

	Age ≥ 50				Age < 50			
	OR	95%CI		*p*-Value	OR	95%CI		*p*-Value
MetS-1 (Yes)	6.38	1.70	23.85	0.006	0.18	0.02	1.74	0.139
Epilepsy-1 (Yes)	3.12	1.15	8.41	0.025	0.40	0.11	1.46	0.166
Age (years)	1.02	0.98	1.07	0.27	1.00	0.95	1.06	0.923
Sex—female	0.98	0.40	2.41	0.967	0.38	0.12	1.19	0.098
Standardized BMI	0.93	0.57	1.51	0.756	0.99	0.53	1.85	0.981

## Data Availability

The original contributions presented in this study are included in the article. Further inquiries can be directed to the corresponding author.

## Supplementary Materials

The following supporting information can be downloaded at: https://www.mdpi.com/article/10.3390/diagnostics15091144/s1, Table S1. Variables of interest for data collection of CCM patients; Table S2. Strong interaction between age and metabolic syndrome (MetS) on hemorrhagic stroke among Hispanic CCM patients; Table S3. Distribution of MetS, epilepsy and hemorrhagic stroke by age groups among Hispanic CCM patients.
