# Supplementary material for: Metabolic Syndrome and Hemorrhagic Stroke in Hispanic Elderly Patients with Cerebral Cavernous Malformations
Source: Diagnostics (Basel). 2025 Apr 30;15(9):1144. doi: 10.3390/diagnostics15091144 (PMC12071915; doi:10.3390/diagnostics15091144)
Supplement: Supplementary file 1 [file diagnostics-15-01144-s001.zip › diagnostics-3451036-supplementary.pdf]

Supplemental Materials

Legends

Suppl Table S1. Variables of interest for data collection of CCM patients. All clinical data from the patients admitted to TTUHSCEP/UMC presenting with confirmed or assumed Cerebral cavernous malformations (CCMs) will be collected based Variables of interest.

Suppl. Table S2. **Strong interaction between age and metabolic syndrome (MetS) on hem orrhagic stroke among Hispanic CCM patients**

Suppl. Table S3. **Distribution of MetS, epilepsy and hemorrhagic stroke by age groups among Hispanic CCM patients**

Suppl Table S1A. Variables of interest for data collection of CCM patients. All clinical data from the patients admitted to TTUHSCEP/UMC presenting with confirmed or assumed Cerebral cavernous malformations (CCMs) and/or Inflammatory bowel disease (IBD). will be collected based Variables of interest listed below

**Gender.**
**Age years (EX: 32)**
**Date of first-ever ICH (if occurred) (year)**
**Date of the first-ever seizure (if occurred) (years)**
**Time point of any treatment (neurosurgery, radiosurgery) (if occurred) (year)**
**Confirmed familial disease (MRI pattern plus one affected relative or pos. genotyping) (yes/no)**
**Assumed familial disease (MRI pattern without an affected relative/genotyping) (yes/no)**
**Time point of the last follow-up at the hospital/institution (Year)**
**Confirmed mutation (CCM1/2/3) (if available)**
**Functional state (mRS) at last FU (if available)**
**Stroke Type (Ischemic 1 Hemorrhagic 2)**
**Place of Birth (Mexico, El Paso, UNK)**
**Admit Date (Year only)**
**Hypertension (1, yes; 2, no)**
**Hypertension numbers**
**Systolic BP at presentation**
**Diastolic BP at presentation**
**Mean Arterial Pressure number**
**Tobacco abuse (1, yes; 2, no)**
**Tobacco Info (packs/day)**
**Diabetes(1, yes; 2, no)**
**Diabetes Info (I/II; glucose Value)**
**Heart Disease (1, yes; 2, no)**

Suppl Table S1B. Variables of interest for data collection of CCM patients. All clinical data from the patients admitted to TTUHSCEP/UMC presenting with confirmed or assumed Cerebral cavernous malformations (CCMs) and/or Inflammatory bowel disease (IBD). will be collected based Variables of interest listed below.

ICD-9 codes	ICD-10 codes	Code description
228.02	D18.02	Hemangioma of brain and intracranial structures
747.81	Q28.3 or Q28.2	Cerebral malformations or Cerebral Arteriovenous malformations
747.82	Q27.9	Congenital Vascular Malformation
747.89	Q28.8	Cavernous malformation of Spinal cord
228	D18.00	Hemangioma of Spinal Cord
228.09	D18.09	Hemangioma of Central Nervous System
742.9	Q04.9	Congenital malformations of brain
348.9	G93.9	Malformation of brain including Brain lesion/mass
430	I60.9	Subarachnoid hemorrhagic stroke
431	I61.9	Intracerebral hemorrhagic stroke
432.1	I62.00	subdural hemorrhagic stroke
432.9	I62.9	intracranial hemorrhagic stroke
345	G40.A01 or G40.A09	Generalized nonconvulsive epilepsy, without mention of intractable epilepsy
345.01	G40.A11 or G40.A19	Generalized nonconvulsive epilepsy, with intractable epilepsy

Suppl. Table S2

Parameters	OR	95%CI		p-value
MetS	0.16	0.03	1.03	0.053
Age-years	1.00	0.99	1.01	0.909
MetS and age	1.04	1.01	1.07	0.007
M	0.35	0.05	2.29	0.35
2.agegroup2	0.92	0.59	1.44	0.05
MetS and age>=50	6.12	0.90	41.60	0.064

Suppl. Table S3

Parameters	Age ≤35	Age (35-50)	Age≥50	p-value
Sample size (N)	33	44	107	
MetS				0.38
0 (No)	31 (93.9%)	37 (86.0%)	86 (83.5%)	
1 (Yes)	2 (6.1%)	6 (14.0%)	17 (16.5%)	
Epilepsy				0.77
0 (No)	24 (72.7%)	35 (79.5%)	79 (74.5%)	
1 (Yes)	9 (27.3%)	9 (20.5%)	27 (25.5%)	
Stroke				0.60
0 (No)	23 (69.7%)	29 (65.9%)	64 (60.4%)	
1 (Yes)	10 (30.3%)	15 (34.1%)	42 (39.6%)	
Hemorrhagic Stroke	OR, p-value	OR, p-value	OR, p-value	
MetS-1 (Yes)	0.41, 0.575	0.44, 0.405	4.67, 0.006	
Epilepsy-1 (Yes)	0.65, 0.606	1.01,0.987	2.37,0.054	

RR: relative risk; OR: Odds ratio
